# Practicality of Acute and Transitional Care and its consequences in the era of SwissDRG: a focus group study

**DOI:** 10.1186/s12913-019-4220-0

**Published:** 2019-06-13

**Authors:** Tenzin Wangmo, Yvonne Padrutt, Insa Koné, Thomas Gächter, Bernice S. Elger, Agnes Leu

**Affiliations:** 1 Institute for Biomedical Ethics, University of Basel, Bernoullistrasse 28, 4056 Basel, Switzerland; 2 Faculty of Law, University of Zurich, Zurich, Switzerland; 3 Center for Legal Medicine, University of Geneva, Geneva, Switzerland; 4 Department Health Sciences, Kalaidos University of Applied Sciences, Zurich, Switzerland

**Keywords:** Acute and transitional care, ATC, DRG, Switzerland, Health policy, SwissDRG

## Abstract

**Background:**

Switzerland recently introduced Acute and Transitional Care (ATC) as a new financing option and a preventive measure to mitigate potential side effects of Swiss Diagnosis Related Group (SwissDRG). The goal of ATC was to support patients who after acute treatment at a hospital require temporary increased professional care. However, evidence is lacking as to the practicality of ATC.

**Methods:**

Using qualitative focus group methodology, we sought to understand the implementation and use of ATC. A purposive sample of forty-two professionals from five Swiss cantons participated in this study. We used a descriptive thematic approach to analyse the data.

**Results:**

Our findings first reveal that ATC’s implementation differs in the five cantons (i.e. federal states). In two cantons, only ambulatory variant of ATC is used; in one canton only stationary ATC has been created, and two cantons had both ambulatory and stationary ATC but preferred the latter. Second, there are intrinsic practical challenges associated with ATC, which include physicians’ lack of familiarity with ATC and its regulatory limitations. Finally, participants felt that due to shorter hospital stays because of SwissDRG, premature discharge of patients with complex care needs to stationary ATC takes place. This development does not fit the nursing home concept of care tailored to long-term patients.

**Conclusion:**

This empirical study underscores that there is a strong need to improve ATC so that it is uniformly implemented throughout the country and its application is streamlined. In light of the newness of ATC as well as SwissDRG, their impact on the quality of care received by patients is yet to be fully understood. Empirical evidence is necessary to improve these two measures.

## Background

Enabling careful transition of patients from acute care hospitals to other care settings is important to ensure continuity of care, reduce re-hospitalization [1–3], and save costs [4]. Transitional or intermediate care ensures that patients receive necessary support at another setting, such as specialized nursing homes, rehabilitation centers, hospitals with intermediate care facilities, or at home with ambulatory nursing care [4–6]. Naylor and colleagues [7] identified key components of comprehensive transitional care, which include educating and engaging patients as well as caregivers, addressing their well-being needs, appropriately managing medications and complex health needs, ensuring care continuity, and accountability. Several studies evaluating transitional care interventions highlight their positive outcomes, such as reduced readmission and decreased mortality [4, 8–11].

In Switzerland, the Federal Law on Health Insurance (Bundesgesetz über die Krankenversicherung (KVG)) introduced Acute and Transitional Care (ATC) with a special financing option in 2011 (Art. 25a para 2, KVG, SR 832.10). The goal was to support those patients who, after acute treatment at a hospital, require increased professional care for a limited amount of time. ATC aims to allow patients to restore to the health status they had before hospitalization and facilitate their return home. According to Art. 25a (para 2) of the Federal Law on Health Insurance, a number of requirements must be fulfilled for the canton (i.e. federal state) and health insurance to participate in its financing [12, 13]. Specifically, there must be a necessity of using this discharge option, it should follow hospital stay, and a hospital doctor should prescribe it. Differently from rehabilitation after hospitalisation, prior cost-approval by health insurance is not required for ATC [12, 14, 15]. According to the law, different care providers can offer ATC, namely nursing care professionals, home care services, and nursing homes. The first two offer an ambulatory variant of ATC, whilst nursing homes provide stationary ATC. This difference has cost-related consequences for patients because the stationary variant comes with room and board costs [12].

ATC is meant to be financed on a dual-fix basis by the canton and the insurer. That is, the canton pays at least 55% of the cost and the insurer 45%. However, according to the law, it is unclear which costs are to be reimbursed and by whom [16]. Thus, the reimbursement is implemented variably in the different cantons. As a rule, the cost of care is reimbursed, while the remaining cost associated with nursing home room and board are paid by the patient [12].

ATC aims to support patients who may be released early from the hospital because of the implementation of Swiss Diagnosis Related Group (SwissDRG). At an international level, DRG is associated with earlier discharge of patients, thus raising concerns related to poorer patient outcomes [17], but such alleged negative side-effects of DRGs are yet to be substantiated in Switzerland [18–22]. A worldwide systematic review of DRG outcomes [23] and a scoping review examining DRG in Switzerland and Germany [24] underlined that neither positive nor negative conclusions could be drawn from available data in light of its heterogeneity. However, it is widely believed that DRGs result in worse quality of care for vulnerable patient groups [15, 25]. It is thus crucial that ATC is implemented appropriately at a practical level, so that potential negative outcomes of DRGs are mitigated. Interviews with hospital experts in Switzerland revealed that there is scepticism concerning the usefulness of ATC [26]. Furthermore, it is feared that certain groups of vulnerable patients - in particular older persons, children, and those who lack language competency – are likely to be discriminated in a framework where the hospital payment system is based on DRGs [13, 27].

Because ATC is a relatively new discharge option, we know little about its implementation and actual use. As part of a nationally funded project on ATC, we gathered both quantitative and qualitative data to understand this discharge option. Our first findings from the quantitative part of the project revealed that more problems occurred with patients who were discharged to ATC when compared to other groups (including rehabilitation), and that female patients were more likely to receive ATC as a discharge option in comparison with rehabilitation [28]. In another paper, we explored medical records of patients and found that older patients lacking private insurance (supplementary to mandatory basic health insurance) and having smaller social networks were more likely to receive ATC than rehabilitation [29]. Therefore, our previous results reveal that vulnerable patient groups (i.e. older female patients and lacking private insurance) were discharged to ATC, an undesired option considering the additional nursing home costs. That certain vulnerable groups tend to receive ATC raises questions on detailed planning of hospital discharges. In light of the very limited empirical evidence on the practicality and the implementation of ATC, we used qualitative focus group methodology to explore professional stakeholders’ perspectives on the implementation of ATC.

## Methods

### Participant selection

The target participants of our focus groups were from five of the 26 cantons in Switzerland. They were selected to represent three different language regions: German-speaking (three cantons), French-speaking (one canton), and Italian-speaking (one canton). The five cantons were selected purposively but their selection was aimed at ensuring representation of the three main language regions in the country and a good mix of urban and rural cantons. Because the regulation was unclear and based on previous criticism [26], it was important to examine cantonal differences in its implementation. Focus group participants included professionals (a) directly engaged in discharge management of patients after their hospital stay, (b) providing care for patients requiring ATC, or (c) working in this context but not providing direct hands-on care. More specifically, we contacted professionals who were providing discharge support in hospitals, healthcare providers offering home care services (known as spitex), directors of nursing homes, representatives from patient rights organizations, and other professionals involved in the delivery of ATC. The study received the approval of the cantonal ethics committee Zurich.

### Participant recruitment

Target participants’ recruitment occurred via purposive sampling, that is, we searched the internet for experts who would fit our target group and as some members of the research team were familiar with the healthcare field, we used personal contacts to reach appropriate participants. Moreover, potential participants themselves also suggested other professionals from their respective cantons that they deemed valuable for our study (snowball sampling). Once we had compiled a list of possible participants, a member of the research team contacted them. The email to the prospective study participants contained information about the study and inquired about potential interest in participating in a focus group discussion. If interested, contacted professionals selected dates (and times) for the focus group session using a Doodle link. A research team member followed-up with all professionals who did not answer the email by contacting them via phone.

### Study participants

Out of the ninety-one professionals contacted to partake in this study, 42 agreed to participate on the selected date (response rate 46%). Among those who participated in this study, 14 (33%) were men and 28 (67%) women. The participants were from the following professional groups: physicians, nurses, social workers, lawyers, and administrators. They were working in hospitals (*n* = 16), nursing homes (*n* = 8), home care service (*n* = 8), administrators from local health departments (*n* = 5), private practice (*n* = 2), hospice (*n* = 2), and patient group organization (*n* = 1). Participants’ consent for recording the discussions were sought and at the time of the focus group discussion, they were reassured that all information gathered would be treated confidentially (i.e. research team would ensure that participants could not be identified individually once results would have been published).

### Interview guide

The study team developed an open-ended focus group discussion guide to help deliberate the flow of the communication occurring within the group. Our discussion questions were: (a) What services are available to patients who at the point of discharge from hospitals require nursing care? (b) What are the advantages and disadvantages of the different discharge options? (c) What does the new regulation on ATC mean to them? (d) What limitations do they see / experience in the implementation of ATC? (e) What challenges and needs do they view in the implementation of ATC? Probing questions were necessary.

### Focus group interview procedures

We organized seven focus group discussions in five cantons. During all focus groups, two researchers (YP and/or BA) were present who were familiar with the study and the focus group methodology. YP is a PhD student completing her doctoral education on the topic of ATC and BA is a sociologist by training and supported data collection. Since interviews were carried out in three languages (German, French, or Italian), we sought support of other team members (IK and EdC) for one focus group each. IK is a trained physician and knowledgeable on research methodology. She carried out one focus group discussion with BA. EdC is a post-doc trained in empirical ethics and supported as the note-taker with YP during another focus group.

During the focus group, one researcher (YP or IK) led the focus group discussions, while the other (BA or EdC) made notes, which helped during the transcription phase to identify the speakers. These notes provided summary thoughts concerning the content of the discussions. The length of the discussions ranged from 61 to 111 min (average 80 min) and the number of participants varied from three to 12. Focus groups took place at a venue that all participants could easily reach. Only two discussions were video recorded and the remaining five audio recorded because transcribing video recorded focus groups turned out to be more difficult and time consuming than transcribing audio recordings without providing significant additional information. As none of the study participants requested to see their transcripts, we did not return the transcripts to the study participants.

### Data analysis

TW carried out the first analysis of the transcriptions using a descriptive thematic approach. This method was applied since the research team was interested in understanding the entire process beginning from discharge and related hurdles described by the study participants [30]. Significant themes identified during this first analysis were discharge options and their underlying criteria; challenges associated with each discharge option; and recommendations to improve post-hospital care. All authors received copies of this first analysis. We held a coding meeting to discuss the main themes (discharge options, criteria, challenges, and recommendations) and their identified relevant sub-themes. After our initial discussion, the authors decided to delve only into the problems related to the use of ATC as the discharge option. In light of this targeted goal, using the analysis done above, three authors [TW, IK, YP] retrieved only the relevant codes and their corresponding coded segments for further re-analysis. We used MAXQDA.18 to manage the coding process.

All authors checked and agreed upon the study results and their interpretations presented in this paper. Finally, three authors (TW, IK, YP) translated the quotes from original language to English and one author (AL) fluent in all languages checked these translations. Quotes (presented in Tables) have been supplemented with addition information in […] where necessary for clarity and (…) indicates that text was edited that did not add any additional information to the quote.

## Results

Concerning the use of ATC, the following three themes were important that underpin ATC’s (lack of) practicality and its consequences for the nursing homes: (a) limited implementation of ATC including its differential implementation; (b) challenges with ATC as a discharge option and its reduced value in practice; and (c) the needs of complex patients that nursing homes offering ATC are unprepared to address (see Table 1 for themes and sub-themes).

**Table 1:** Use of ATC as a discharge option

Theme	Limited implementation (of ATC)	Challenges associated with ATC	Needs of complex patients
Sub-theme 1		Lack of familiarity with ATC	Nursing home concept does not fit ATC patients
Sub-theme 2		Additional administrative burden	Nursing homes are not prepared to care for complex health needs
Sub-theme 3		Inherent limitations of ATC regulation: (a) 14 days and (b) cost issues	

### Limited implementation

Since ATC can either be provided as an ambulatory form through home care services or as a stationary care in nursing homes, its implementation varies between cantons. In one canton (Italian speaking), ATC is offered only in its stationary variant. Participant P4 (FG7) asked “But I wanted to ask something if maybe someone knows, [whether] ATC home in [name of the canton] does not exist or exists (…)”, to which participant P2 (FG7) replied “It was a considered political choice not to allow ATC at home.” In two cantons (both German-speaking) ATC is mostly provided in its stationary variant, with ambulatory ATC provided only when needed. However, in two other cantons (one German- and one French-speaking) ambulatory ATC is the only option available for patients: “no, actually there is no possibility to offer [stationary] ATC … we do not have any nursing home which offers that” (P2, FG1). Another participant added: “Well in [name of the canton] the stationary division is not implemented ... only the spitex [home care service providers] has the mandate to provide [ATC]” (P2, FG2).

In the German-speaking canton where only ambulatory ATC is available, participants revealed that patients in need of nursing care after hospitalization – but unable to return home with home care support – were sent to rehabilitation centers. On the contrary, participants from cantons where stationary ATC is available discussed that the number of places in nursing homes that take ATC patients is limited or that there are designated nursing homes specifically for ATC patients (see Q1, Table 2). According to the focus groups, however, this did not mean that all patients in need of ATC are able to obtain a place in these institutions. Unavailability of places for ATC care made rehabilitation a preferred discharge option, also because of the reduced economic burden for the patient (see Q2, Table 2).

**Table 2:** Participants’ quotes for theme “Limited Implementation”

Quote (Q)	Quote from participants
Q1	Yes, it is an effort really, we have 48 h during which we must admit patients coming from the city hospitals. Sometimes we have one bed available and eight registrations. That means we have to move things [make difficult decisions]. (…) (P5, FG4)
Q2	Participant: But nursing home charge [ATC patients], 180.- for the room and board [per day] while the nursing care cost of 21.70- is deductible. (P1, FG1) Moderator: Yes, that’s probably the same cost in our city (…) this is a couple of thousand Swiss francs, which the patient then has to pay (…) Participant: That’s why rehabilitation is still the more interesting alternative. (P3, FG1)

### Challenges with ATC as a discharge option

#### Lack of familiarity with ATC

As hospital physicians are the authority responsible for recommending ATC and signing off on the ATC request, many participants underlined that physicians’ lack of knowledge about ATC was a hurdle: “I remember that recently I more or less assigned a patient there [ATC] and the doctor told me:” “What is this? I have no idea” (P4, FG1).

Furthermore, several doubts emerged concerning the differences between ATC and other post-hospital services routinely provided to patients. In particular, it was unclear how - from the perspective of patients - (ambulatory) ATC provided through home care service is different from other regular home care services (see Q3, Table 3). Also, criteria why patients should be sent to a nursing home for ATC and not rehabilitation were hazy. Thus, the medical benefits in prescribing ATC were largely unclear (see Q4, Table 3).

**Table 3:** Participants’ quote for theme “Challenges with ATC as a discharge option”

Quote (Q)	Quote from participants
Q3	(...) it’s more work to do ATC [instead of regular spitex (i.e. home health service provider]], frankly, apart from the added value for the patient who doesn’t pay for 14 days the eight francs, right, but all the work hospital doctors have … it’s an administrative tedium, for finally, well, actually, the patient saves 14 times eight francs. (P1, FG5)
Q4	ATC plays a very small role. Well, I remember when they established it, spitex made an event and informed us, what it is and this caused astonishment, because the key question was whether there is a new offer? And, well I assume spitex does what it can. That means from the supply end, it was not plausible that anything was done differently or more was done than before. But if one then calculates where the benefit for whom lies, it was only this nursing care deductible. (P2, FG2)
Q5	We do the registration [for Spitex] electronically … [which], takes two or three minutes administratively, then it is done and sent out. … And for ATC, it looks like, for now they’re asking us to fill in their forms [in addition to the previous information that must be sent], and we must give the same information again. (P2, FG2)
Q6	This is of course what case management does for us, the physician signs it. (…) I would never have come up with the idea of presenting this to a physician, for me it is quite clearly a nursing order, so in my view, the physician cannot do that. (P4, FG1).
Q7	ATC is limited in time, to actually 2 weeks, which, I think, can be extended, I think the average stay is 3 weeks, then the financing simply changes, which then changes to a temporary bed. [...] But the killer criterion is the [limited] time. (P2, FG6).
Q8	And then there are those that are ... the acute transitory care where patients must have the requirements: age AVS [legal age of retirement], therefore 64 for women, 65 for men. An estimated ATC of 14 days where there is the possibility of extending the stay (…) that gives additional 14 days then for a total of 28 days. (P1, FG7)
Q9	It’s true that 14 days are rather short, that is the maximum duration of ATC. Extending ATC to 30 days would not mean to prescribe 30 days for all. It would mean, if 16 days were appropriate, you could give 16 days. It is the moment to assert that 1 month of ATC would be more facilitating than 14 days ATC. (P1, FG5)
Q10	We get those people from the ATC and we first need to find out: What is the goal for that person? What goals does she have how it’s supposed to work at home? Be it taking two steps on the stair or walking around with support. And 2 weeks is simply very short. People often come in a very acute phase. […] (P4, FG2)
Q11	We also always tell that it may not be realistic, the 2 weeks. So with me there is no cheating package by saying after 2 weeks they are fit again and at home afterwards. I rather say that can be 3, 4, 5, 6 weeks.. But it’s also for the patients – I experience sometimes – that they would rather go there [ATC], because they don’t want to stress themselves as for example in a Reha, where there is quite a program to follow within 2 weeks. But [as a patient] I have more time and can relax/recover there with the option – that is my goal – to go back home afterwards. (P3, FG3)
Q12	Yes, well [the cost information] must be told [to the patients] in the hospital, because that sums up extremely and the relatives are relieved when the patient goes to rehabilitation because that is a lot cheaper... That is an aspect, an important one. (P4, FG1)

#### Additional administrative burden

Related to the issue of not completely understanding the ‘purpose’ of ATC and viewing it as an economically ‘non-beneficial’ option, several participants pointed out the additional paper work that ATC requires (see Q5, Table 3). Moreover, they complained that this discharge option has to be signed off by the physician, who may not be in the most appropriate position to evaluate the post-hospitalisation needs of patients (see Q6, Table 3).

#### Inherent limitations of ATC

The first inherent limitation that all participants noted with respect to ATC as a discharge option was the 2 weeks for which it can be prescribed. They felt that this arbitrary time limitation is a strong disincentive to prescribe and/or use ATC. Participants also underlined that ‘2 weeks’ is not only an unrealistic time-window to achieve complete recovery (see Q7, Table 3), but also insensitive to the potentially longer care needs of patients particularly those who are older. For one of the five cantons, participants reported that there is the possibility to extend ATC by 14 additional days to a total of 28 days when patients have reached retirement age (see Q8, Table 3). Moreover, participants agreed that it is a misguided expectation that patients would be able to return to their pre-hospitalization health status within the 2 weeks of ATC. This two-week time limit is inadequate in light of many things that need to be done with patients to ensure they can actually achieve the objective set out underlying ATC, namely that of patients ‘being healthy’ enough to return to the ‘normal life’ (see Q9 and Q10, Table 3). Very few participants thought that although ATC is time limited, this is still a useful discharge option since it provides the ‘breathing’ time for the patient to not feel rushed to become healthy (see Q11, Table 3).

The second limitation of ATC was the costs of this discharge option. In almost all focus groups, participants discussed how ATC was a legal way of moving financial burden from one party to the other. For instance, one participant, P5 (FG4) expressed **“**But actually the health insurance makes a good, nice break where the insured has to pay himself and then they pay.” In fact, in the cantons where stationary ATC is offered, patients need to pay for the room and board cost of the institution where they receive ATC, an information which needs to be disclosed to the patients (see Q12, Table 3). Only patients in the Italian-speaking canton pay a smaller contribution (compared to patients in other cantons) per day towards stationary ATC instead of the entire room and board costs. Along these lines, participant P3 (FG7) stated, “The patient pays CHF 50 per day (…) The health insurance pays a lump sum of CHF 125, nursing care, medical care, laboratory, physiotherapy, all-inclusive equitable drugs [in addition to what is paid by the canton and the local community]”.

### The needs of medically complex patients

Participants reported that because of the implementation of SwissDRG, consciously or even unconsciously, hospital stays have shortened, and that patients with complex care needs are discharged prematurely. The category of medically complex patients included not only those with multiple illness (typical of older patients), but also those patients (e.g. with mental illness or other challenging medical needs) for whom the post-hospital settings (i.e. nursing home) was deemed unsuitable. This raised discussion among participants whether the discharge option of ATC fits the concept of care offered by nursing homes and their general unpreparedness not only for the medically complex situations that ATC patients might present, but also for administrative burdens associated to ATC (see Q13, Table 4).

**Table 4:** Quotes for theme “The needs of medically complex patients”

Quote (Q)	Quote from participants
Q13	I think it’s a bit delicate because geriatric centres and long-term care [nursing homes] receive every type [of patients]. Well we have a lot of psychiatric [patients]. Then from the hospitals too, we get all [types of patients], complex oncological radiotherapy and chemotherapy-break, accident surgical cases, (…) Then we also have palliative transfers… (…) and we just have every type [of patients] and that makes it so difficult. (P4, FG4)
Q14	After all, most nursing care facilities are geared towards long-term care. Out of history, out of tradition. Whether there really is the idea of curing people, mobilizing them, letting them become independent in activating care and what other concepts there are. It’s a bit of a question mark for me. I’m not so sure. [if nursing homes have these goals]. (P1, FG4)
Q15	The nursing homes are basically geared towards long-term care in terms of their nature, their facilities, their infrastructure. (...) And of course such a temporary stay has completely different requirements. We don’t do ATC. But the temporary residents have completely different demands, they want a furnished room, their goal is to get well and home as soon as possible, that is their goal. (P3, FG6)
Q16	And I see, when I do the review of the medical reports, I get registrations, where I first need to organize all the equipment. Then we have a tracheostomized patient, who we need to aspirate, may need artificial respiration and sometimes need monitoring because they are still very unstable. Then I have to organize all the material, then we need to have trainings on the handling/use of those equipment, and have to train the personnel. Then we have to think through what we do at night, when the whole house with six stations have only two qualified persons [registered nurses] and the rest are nursing associates. When we then have a tracheostomized to aspirate… yes, how do we manage that? (P4, FG4)
Q17	Well it is really challenging. We have difficulties with reimbursement [from insurance], the expectations for the infrastructure are clearly higher. They want fitted beds like in the hospital with TV and with all the [additional things]. It’s not good for the community [nursing home because], they are here [in nursing home], they leave again [after meeting their health needs]. No, it’s really… The planning security, the economic planning security of the positions in nursing with such a big proportion [of temporary residents] it’s not good, not very good. (P3, FG6)
Q18	If every 14 days someone new is being admitted, who needs to be registered with the whole thing or one has to look at what this person, the resident needs to go back home again. That is a really complex situation. […] One has to do the care plan together with the doctor, together with the resident and so, we have to have very very early a lot of conversations. Because 14 days passes very quickly. So once someone is there, we already start to plan the discharge. (P4, FG3)

#### Nursing home concept does not fit ATC patients

Participants said that nursing homes are not meant to be institutions with a steady turn-over of patients and that they are not in the business to help patients recover and return home. In theory, persons who live there are residents that see the institution as their ‘home’ (see Q14 and Q15, Table 4).

#### Nursing homes are not prepared to care for complex health needs

As reported previously, participants working in nursing homes noted that their setting is not apt to address the needs of patients with complex health situations. They stated that nursing homes provide support for elderly persons who are residents and in need of basic care, and not complex medical assistance. “[In the nursing home] we have so much qualified personnel, the whole structures are oriented at geriatric, very old people and now we have just all [types of patients]” (P4, FG4). Therefore, receiving patients with complex medical needs raise issues at three levels: the need to organize medical equipment that nursing homes do not generally have, the difficulty of nursing home professionals who are trained mostly to care for older persons, and the poor match between complex medical care and personnel availability (see Q16, Table 4).

Thus, accepting ATC-patients decisively influences the workload of nursing homes. This includes additional requirements for insurance reimbursement, extra billing work, having to make care plans for patients whose stay is limited to 14 days, and the need to arrange for equipment that nursing homes do not possess (see Q17 and Q18, Table 4).

## Discussion

This study presents important findings that confirm several concerns raised about ATC. According to the ATC regulation, this discharge option may be offered in two variants: ambulatory or stationary [12, 16]. Our data show that the standards of ATC differ geographically. Specifically, in the five cantons from which we gained information, it was clear that ATC was provided either in the stationary variant only (Italian-speaking) or in the ambulatory variant only (one German-speaking and the French-speaking canton). In two cantons (both German speaking), both variations are used, with a preference for stationary ATC. Although this variability seems to be a result of cantonal differences in applying the ATC regulation, this has consequences for the patient [31]. For example, it emerged that patients in German-speaking cantons where stationary ATC is used have to pay the total room and board costs, while those in the Italian-speaking canton pay only a portion of the room and board costs. Stationary ATC in many cantons is associated with out-of-pocket cost for the patient amounting to approximately CHF 180 per day, except for in one canton, where patients pay CHF 50 per day [31]. The financing of ATC remains sub-optimal [16] and from our interviews, it appears to not be uniform. The reason for this differential implementation of the law requires clarification.

Study participants noted that services provided through the ATC discharge option are not unique, since they do not differ substantially to what is already available outside the ATC framework [e.g. short-term stay at nursing home; nursing care service at home]. Thus, except for changes in the financing structure of nursing homes and home care service for the 14 days during which ATC order is given, there seems to be no qualitative difference in the care patients receive. Furthermore, the legal requirement that ATC has to be prescribed by a hospital doctor [12, 13] was deemed unhelpful. This is because in practice doctors have to complete additional registration forms concerning the need for care. Such a criticism is sound since ATC is designed to support patients with post-hospitalization nursing care needs. Healthcare professionals directly caring for the patient (e.g. nurses, case managers) might be better suited than hospital doctors to assess such needs. Monego and colleagues [16] recommend that general practitioners be authorised - on top of hospital doctors - to prescribe ATC.

Our findings also underlined that 14 days of ATC is a very limited period to achieve the purposes – set out by the law – of restoring patients’ health to pre-hospitalization level and to return home. The regulation does not state the exact number of days for which ATC can be prescribed, but defines that financing is limited to 2 weeks. In case a patient is discharged to rehabilitation, although the insurer needs to approve the cost prior to admission, this discharge option can be prolonged after its initial 14 days upon the request of the physician. Only the Italian-speaking canton provided longer ATC, but exclusively for patients of legal retirement age. That ATC should be prescribed for longer period and its financing should be better structured have been suggested repeatedly [12–14, 16, 25], and our empirical evidence strongly supports these claims.

For Switzerland, evidence is lacking to substantiate the fears that patients are actually released earlier and with poorer health status [13, 26] due to DRG implementation [18, 32, 33]. However, several of our study participants stressed how nursing homes have to cater for patients with growing medical complexity, hints at possible early discharges because of SwissDRG. Furthermore, the fact that certain nursing homes must accept ATC patients mean additional work for these institutions, including frequent intake-and-release related paperwork, managing the financing structure of ATC, and figuring out how to adjust to the varying medical situations. Since nursing homes traditionally care for older adults who become long-term residents – and for whom they provide mostly assistance with activities of daily living, the challenges our participants noted are somehow not surprising. Having to admit growing numbers of short-time patients who require a higher workload, but whose reimbursement occurs on the same financial basis as other long-term residents, might indeed degrade quality of care for long-term residents.

Although ATC shares commonalities with transitional or intermediate care available in the UK and the US [3, 6, 11], differences lie in the fact that ATC is a discharge option with specific financing and another institution provides the service. The latter element entails that continuity of personnel in the patients’ care is lacking. Another factor worth mentioning is that our participants did not discuss the educational component - in the sense of patient and caregiver empowerment to address medical care – which is often highlighted in the literature [1, 8, 9]. The only exception in this respect was the expressed need that patients and family members receive information about the cost associated with stationary ATC. As noted earlier, cost issues must be clarified at the legal level so that ATC is financed equally nationwide [16].

### Limitations

As a qualitative focus group study, our findings are not generalizable, since we recruited participants from five of the 26 Swiss cantons. Nevertheless, we deem our results to be important for they reveal empirical evidence that highlight the unclear nature of ATC regulation [12–14, 25]. Even though our study participants belonged to different professional backgrounds, they presented similar concerns underlying common challenges in implementing ATC. We did not include patients’ and their caregivers’ perspective in this paper, a shortcoming of our focus group methodology. Another limitation could be social desirability, that is, participants may have reported what they felt the researchers would like to hear. Since the discussions took place within a focus group, it could also be that those with diverging views did not disclose their opinions and agreed with the issues reported by their colleagues. Finally, as data collection occurred in three languages and quotes used in this manuscript are in English, we cannot exclude errors during this process.

## Conclusions

Our findings underline that a number of issues such as the financing of ATC, its limited duration, and the medical responsibility (i.e. who can prescribe it) need review at the policy level and, potentially, a revision of the existing regulation. Clarifying these concerns will ensure uniform and correct implementation of ATC across the country. As evident in previous research [28, 29] vulnerable patient groups, such as older women and those lacking supplementary insurance, tend to be disproportionately discharged to ATC. A standardized assessment of patients’ needs is critical so that ATC and other discharge options (e.g. rehabilitation) are prescribed uniformly. Such practice will assure equal access to different care options after hospitalization and avoid burdening certain patient groups. Beyond that, there is need for further research to (a) evaluate if ATC is indeed a measure to protect vulnerable patient groups, (b) alleviate the dangers of precocious hospital discharge, and (c) explore how its use is being justified in light of stationary ATC’s cost burden.

## Data Availability

The datasets used and/or analysed during the current study are available from the corresponding author on reasonable request.

## Abbreviations

ATC: Acute and Transitional Care
DRG: Diagnosis Related Group
KVG: Bundesgesetz über die Krankenversicherung (i.e. Federal Law on Health Insurance)
SwissDRG: Swiss Diagnosis Related Group
